# The application of One Health concept to an outdoor problem-based learning activity for veterinary students

**DOI:** 10.14202/vetworld.2016.955-959

**Published:** 2016-09-10

**Authors:** T. A. Tengku Rinalfi Putra, Mohd Noor Mohd Hezmee, N. B. Farhana, H. A. Hassim, A. R. Intan-Shameha, I. H. Lokman, A. Yusof Hamali, M. S. Salisi, A. A. A. Ghani, M. S. Shahudin, M. A. L. Qayyum, A. Hafandi, R. Speare, S. G. Fenwick

**Affiliations:** 1 Department of Veterinary Preclinical Sciences, Faculty of Veterinary Medicine, Universiti Putra Malaysia, 43400 Serdang, Selangor Darul Ehsan, Malaysia; 2 College of Public Health, Medical and Veterinary Sciences, James Cook University, Townsville 4811, Australia; 3 Department of Infectious Disease and Global Health, Cummings School of Veterinary Medicine Tufts University, Greater Boston, Massachusetts, USA

**Keywords:** Doctor of Veterinary Medicine, One Health, problem-based learning

## Abstract

**Background:**

The One Health (OH) approach, which seeks to bring together human and animal health, is particularly suited to the effective management of zoonotic diseases across both sectors. To overcome professional silos, OH needs to be taught at the undergraduate level. Here, we describe a problem-based learning activity using the OH approach that was conducted outdoors for 3^rd^-year veterinary students in Malaysia.

**Materials and Methods:**

A total of 118 students, divided into two groups, completed the activity which spanned 1½ days at a deer park adjacent to a wilderness area. Students were asked to evaluate the activity using an online survey that had quantitative and qualitative components.

**Results:**

Response rate was 69.5%. The activity was rated excellent by 69.5% and good by 30.4%. Levels of satisfaction were high on a range of criteria. 97.5% of students intended to take action in their studies as a result of what they had learned.

**Conclusions:**

Delivery of an outdoor problem-based learning activity using OH approach was very successful in terms of participation, knowledge delivery and understanding, and the willingness of students to integrate OH into their future practice. For the improvement of future programs, the involvement of other disciplines (such as Medical, Biology, Biotechnology, Biomedical, and Public Health) is being considered.

## Introduction

The Veterinary Problem Investigation course taught in the Faculty of Veterinary Medicine, Universiti Putra Malaysia (UPM), is entirely conducted using the student-centered learning (SCL) and problem-based learning (PBL) approaches [1-3]. Ever since its introduction in the Doctor of Veterinary Medicine (DVM) program in this university, the course was conducted in the classroom. The students have not had the first-hand opportunity in exploring, investigating, and experiencing real-life scenarios (experiential learning) related to veterinary issues that they may face later on after graduation. Moreover, students at this stage have not been exposed to a systematic way of tackling veterinary problems using an integrated approach or concept such as One Health (OH).

To address this, we have trialed a new teaching activity with the aim of introducing the OH concept [4-8] and competencies in veterinary problem investigation and management to 3^rd^-year students. They have acquired prior knowledge of animal nutrition, husbandry, production, animal breeding, and basic preclinical sciences during year 1-3 of the program. The activity also provided a platform for “experiential learning “among students who were previously accustomed to solving veterinary problems in the classroom settings. Activities were planned to enable students to appreciate the complexity of the cases by first-hand observation, conduct onsite discussion, and plan a successful management protocol to curb problems related to this farm that involved OH competencies such as management, systems thinking, to nurture students’ soft skills such as the ability to work in a team, support other group members by sharing knowledge, time, and ideas in solving particular problems (values and ethics), promote the element of self- and team-trust, ability to identify and resolve issues and conflicts with proper actions in a professional manner (communication and informatics) [9,10], and to establish ties between the faculty/university and the industry (collaboration and partnership).

By the end of this exercise, the students were expected to learn and describe the role of good animal husbandry, production practices, biosecurity, welfare and management of wildlife in captivity, to apply and relate the basic anatomy, physiology, pharmacology, animal production, ethology, welfare and genetic/breeding knowledge in disease/veterinary problem investigation, to discuss the role of wild animals and environment in transmission of diseases and the importance of public awareness and economic impact of wildlife zoonosis, to appreciate the importance of integrating the OH concept in veterinary disease/problem investigation and management, and to implement/exercise the OH competencies acquired for disease investigation and management.

## Materials and Methods

### Ethical approval

Since this was an evaluation to be used to improve the teaching of the activity for the subsequent student cohorts and not a research project, it did not require any human ethics approval.

### Study design

**Figure 1:**
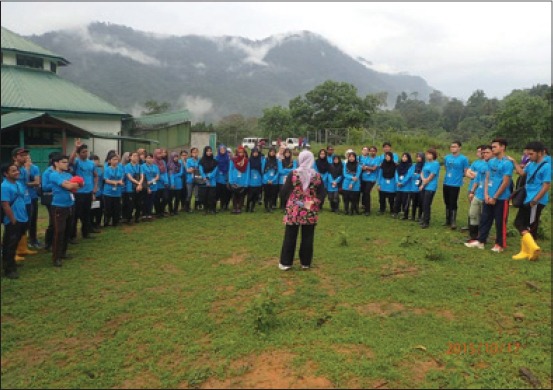
Students being briefed by the program advisor regarding the management in Deer Breeding Centre, Lenggong. Note the Centre adjoins a national rainforest reserve known as the Royal Belum State Park (in background). Picture courtesy of National Coordinating Office, Malaysia One Health University Network.

**Figure 2:**
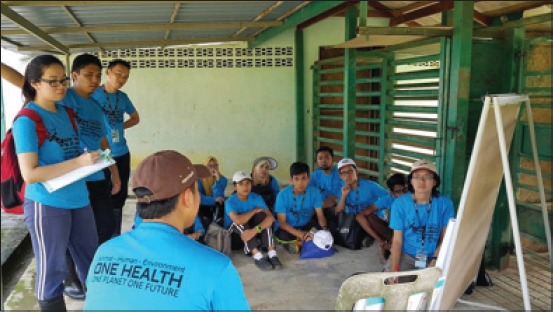
The problem-based learningsession taking place at the deer farm. Picture courtesy of National Coordinating Office, Malaysia One Health University Network.

**Figure 3:**
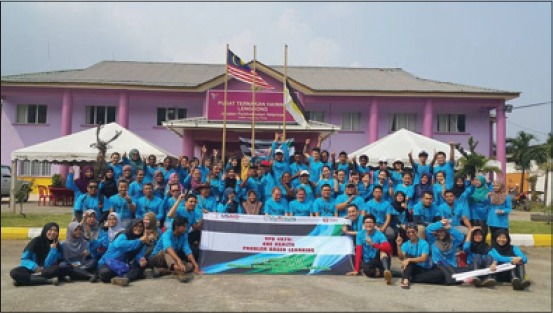
The participants of the program. Picture courtesy of National Coordinating Office, Malaysia One Health University Network.

The entire 118 3^rd^-year DVM students cohort were enrolled as an experimental study group for this exercise. They were divided into 10 groups with 10 to 12 students per group (Figure-1). Each group was assigned 2 facilitators who were well versed in the case designed for this outdoor PBL and also well trained to handle questions and the sessions effectively (Figure-2). The selected location for this outdoor OH-PBL exercise was the Deer Breeding Centre, Lenggong, Perak Darul Ridzuan, Malaysia (Figure-3). The rationale behind the selection of this farm was due to the suitable interaction with the animals reared in the farms, the cases that were designed to be OH-related and the fact that the farm was adjacent to a wilderness area (a classic interface for spillover of emerging infectious diseases from wildlife to livestock) [11]. The students were given two PBL scenarios regarding zoonotic diseases, tuberculosis, and brucellosis, both of which can be transmitted from deer to human [12-17]. This allowed the application of the OH approach for these PBL activities. The case studies were delivered to the students using clues and triggers as described by Lloyd-Jones *et al*. (1998). The students were also introduced to the PBL concept of Facts, Ideas, Learning Issues, and Actions (FILA) table to approach each trigger and clue pertaining to the case given [18]. A total of four triggers were given throughout the activities, and students were required to solve the case using all the information that was available in each trigger using the FILA table. Trigger 1 was given at the beginning of the PBL session, and subsequent triggers were given after all knowledge and information that was extracted from a particular trigger enable the students to move to the next stage of diagnosing the particular case given. The students were required to solve the case and state the definitive diagnosis for the particularcasegiven.

The effectiveness of doing an outdoor OH-PBL for this cohort of students was assessed by an online survey using the Google Docs system to evaluate the activity to allow improvement for future cohorts. The survey had 10 closed questions and several open questions: “Describe what, if any, actions you will take in your work/studies as a result of this course”; “What were the strengths of this course and the activities?”; “What can be done to improve this course and the activities?”; “What single most important lesson did you learn from this course and the activities conducted?,” and “Write any additional comments you may have about this course and the activities.” Responses were voluntary and anonymous.

## Results

Around 82 students completed the online survey, a response rate of 69.5%. Most were female (61 students), making up 74% of respondents. Females made up 77% of the total class.

### Quantitative evaluation

The PBL activity was rated as excellent by 69.5% (57/82) students, good by 30.4% (25/82), and poor by none. Students expressed a high level of satisfaction with many aspects of the activity (Table-1).

**Table-1:** Responses of 3^rd^-year veterinary students to questions about outdoor OH-PBL activity.

Question	Strongly agree (%)	Agree (%)	Disagree (%)	Strongly disagree (%)
The course and the activities met its stated objectives	41/82 (50)	37/82 (45)	3/82 (2.5)	1/82 (1.2)
This course and activities met my expectations	36/82 (43.9)	43/82 (52.4)	2/82 (2.4)	1/82 (1.2)
The course and activities were relevant to my work/studies	47/82 (57.3)	34/82 (41.5)	1/82 (1.2)	0/82
Overall, the speakers at the course and wildlife facilitators were informative	59/82 (72)	23/82 (28)	0/82	0/82
The materials provided were useful	39/82 (47.6)	43/82 (52.4)	0/82	0/82
This course and the activities helped clarify my understanding of “OH”	48/82 (58.5)	33/82 (40.2)	1/82 (1.2)	0/82
The logistics for the course and activities were well managed	45/82 (54.9)	37/82 (45.1)	0/82	0/82
I intend to take actions in my work/studies as a result of what I learned in this course and from the activities conducted	33/82 (40.2)	49/82 (59.8)	0/82	0/82

OH: One Health

The amount of information delivered during the PBL sessions was rated as about right by 95.1% (78/82) and too much by 4.9% (4/82) of respondents.

### Responses to open questions

Written comments from the students provided insight into their opinions on the strength and advantages of this outdoor OH-PBL activity. Examples follow:

“*………It provides new input to the students, where One Health is something familiar but not so known by us. This course has brought more information on the role of myself now and in the future. It also widens my view on One Health, where everyone should be responsible for One Health………*”

“*………It is about the art to solve the cases with given facts on each trigger, which enable us to brainstorm our ideas to generate the possibilities of each given facts. We applied all the knowledge that has been taught to come up with the ideas, learning issues, and the appropriate action that we should take………*”

“*……For me, the strength of this course is that it allowed me to learn things in a fun way. I found it is more effective than learning in a classroom/lecture hall. Moreover, the greenly environment, fresh air, etc., actually quite a good get away from my environment of study………*”

“*………The strength of this course is definitely the fact that it is a very good introductory course to people and community who are still unfamiliar with this “One Health” approach. The facilitators and activities are very friendly, informative, and interactive with the participants. The activities are fairly simple and are able to demonstrate the importance of “One Health” in our surrounding lives………*”

“*………From my opinion, the strength of this course is it applies the knowledge I gain in the class which is theory and when conducted the activities I can apply it in the case………besides that the environment where the activities were conducted is conducive for a student like me………*”

“*………This course introduces one simple concept that assists thestudentto solve problem step by step with helped from experience facilitators. In addition, this course also emphasizes on soft skill which you can get it throughout all activities………*”

These are some of the examples of the written responses by the students in the survey when asked about the strength and advantages of this outdoor OH-PBL program. In summary, the trends of their opinions are mostly centered toward the appreciation of knowledge they have gained from participating in an outdoor PBL, the acknowledgment of understanding the OH concept for the first time and the understanding on aspects of zoonotic disease, and how to manage the situation in cases of an outbreak.

## Discussions

This paper examined whether outdoor PBL can stimulate student interest and boosts their learning motivation and identified aspects of PBL which are engaging to students. PBL is an alternative method to deliver classroom lesson in an SCL environment for both the teachers and the students to understand lessons more and be able to apply the knowledge in a real-life situation. Unconventional evaluation which is genuine to the knowledge setting can have an encouraging authority. Unconventional evaluation quantification might include building up reply items, essays, writing samples, oral presentations, exhibitions, experiments, and/or portfolios [19]. In a PBL classroom, these actions might be much more significant and valid to a problem-solving setting than a customary standardized multiple-choice test. Permitting students to employ in these kinds of actions can allow us to judge key learning issues by assessing and reviewing the students’definite or replicate presentation on major duties [20]. Thus, the best way to introduce a subject matter so that students can fully understand and grasp the knowledge is using a PBL method.

Not all subject matters can be elaborated and formatted to suits the need to use this PBL method. Basic lessons in veterinary medicine such as veterinary anatomy will not utilize the strength of PBL in terms of student’s understanding effectively. A study by Albanese and Mitchell, 1993, has indicated that medical students schooled on PBL performed worse on standardized tests as compared with when performing for clinical tests [21]. This shows that basic lesson such as veterinary anatomy can be formulated as PBL subject, but the outcome may not be beneficial in terms of student performance. Therefore, a clinical approach in designing cases for PBL is warranted.

OH is one of the very important concepts that are currently being championed throughout the world [7,10,22-24]. Recognizing that human health (including mental health via the human-animal bond phenomenon), animal health, and ecosystem health is inextricably linked. OH seeks to promote, improve, and defend the health and well-being of all species by enhancing cooperation and collaboration between physicians, veterinarians, other scientific health and environmental professionals, and by promoting strengths in leadership and management to achieve these goals. The concept has provided the perfect platform for the outdoor PBL in this study to better assimilate the relationship between the real-world cases and experiential learning process. Since the venue for the PBL was in a deer farm where possible real-world scenario of the zoonotic disease can occur, the integration of PBL and OH can be achieved successfully.

A set of questions havebeen posed to the participants in post-program to get the response of the participants toward the effectiveness of the program in terms of their better understanding on the topics; they learned in classroom and the prospective of continuing the same approach in future PBL. A basic question to describe the overall activity has shown that the participants fully appreciate the program as a whole and were happy that the program indeed proves beneficial to them (Table-1). This general question indicates that the participants saw the whole objectives of the program have benefitted them entirely. The participants have also indicated that the overall contents and information in the program were about right and did not exceed the amount deemed too much for them to understand (Table-1). This was in accordance to some PBL practitioners who indicated that too much information in delivering PBL will tend to confuse students more and will put an extra burden to them. The results from the table also indicate that the contents of the material delivered in the program were up-to-date and very relevant to the current situation in the veterinary field. Indeed, the case studies that were used in this PBL-OH program were designed to cater the present climate in zoonotic diseases [25-28]. This information helped the participants to relate the knowledge they learned in the classroom into the actual field scenarios. Meanwhile, the participants also agree that the facilitators in the program have the required knowledge in delivering PBL using the OH model, and this helps them to have confidence and trust during the discussion regarding the zoonotic case studies presented to them. The facilitators were well equipped with the knowledge due to the facts that they were trained efficiently by the University in PBL courses since PBL module is currently being used throughout the campus. In term of OH competencies, they were also trained regularly since the National Coordinating Office of Malaysia OH University Network or MyOHUN is based in the faculty itself and did consistently provide useful training module to all the academic staffs which also served as the facilitators in the program.

## Conclusion

In summary, most of the correspondents agreed that conducting an outdoor PBL in a venue which closely mimic the actual environment of potential outbreak for zoonotic diseases did help the participants to understand and appreciate the lessons more and also provide the impetus for the participants to exercise the knowledge whenever they are faced with a real-life situation in the future. The objectives of programs in delivering an outdoor PBL in tandem with OH have been very successful in terms of participations, knowledge delivery, and understanding and are also able to integrate the life of the current OH practitioners and future OH workers. Limitation of the study was basically due to the time allocated in the program that will be highlighted in future programs. For the improvement of future programs, the team will be discussing more relevant zoonotic diseases with participants and also bring in participants from other health institutions such as medical, biology, biotechnology, biomedical, and public health participants.

## Authors’ Contributions

All authors were involved in running the PBL sessions and data collection. RS, SGF, TATRP, and MNMH wrote and edited the manuscript according to the title. All authors read and approved the final manuscript.

## References

[1] Barrows H. S (1980). Problem-solving learning. Med. Educ.

[2] Tamblyn R. M, Barrows H. S, Gliva G (1980). An initial evaluation of learning units to facilitate problem solving and self-directed study (portable patient problem pack). Med. Educ.

[3] Bastawrous A, Bastawrous M (2011). Problem-based learning, a closer look. Clin. Teach.

[4] Sundberg J. P, Schofield P. N (2009). One medicine, one pathology, and the one health concept. J. Am. Vet. Med. Assoc.

[5] Buttke D. E (2011). Toxicology, environmental health, and the “One Health” concept. J. Med. Toxicol.

[6] Cribb A, Buntain B (2009). Innovation in veterinary medical education: The concept of 'One World, One Health'in the curriculum of the Faculty of Veterinary Medicine at the University of Calgary. Rev. Sci. Tech.

[7] One Health Initiative.

[8] Kahn L. H, Kaplan B, Steele J. H (2007). Confronting zoonoses through closer collaboration between medicine and veterinary medicine (as 'One Medicine'). Vet. Ital.

[9] Lueddeke G, Kaufmann G, Kahn L, Krecek R, Willingham A, Stroud C, Lindenmayer J, Kaplan B, Conti L, Monath T, Woodall J (2016). Preparing society to create the world we need through 'One Health'education. South Eastern Eur. J. Public Health.

[10] One Health Commission.

[11] Jones K. E, Patel N. G, Levy M. A, Storeygard A, Balk D, Gittleman J. L, Daszak P (2008). Global trends in emerging infectious diseases. Nature.

[12] Thoen C. O, Steele J. H, Kaneene J. B (2014). Zoonotic Tuberculosis: *Mycobacterium bovis* and Other Pathogenic *Mycobacteria*.

[13] One Health Initiative Tuberculosis Video.

[14] Rabinowitz P. M, Conti L. A (2010). Human-animal Medicine: Clinical Approaches to Zoonoses, Toxicants and Other Shared Health Risks.

[15] Pappas G, Akritidis N, Bosilkovski M, Tsianos E (2005). Brucellosis. N. Engl. J. Med.

[16] Pappas G, Papadimitriou P, Akritidis N, Christou L, Tsianos E. V (2006). The new global map of human brucellosis. Lancet Infect. Dis.

[17] Waters W. R, Palmer M. V (2015). *Mycobacterium bovis* infectionof cattle and white-tailed deer: Translational research of relevance to human tuberculosis. ILAR. J.

[18] Hmelo C. E, Guzdial M, Edelson D. C, Domeshek E. A (1996). Of black and glass boxes: Scaffolding for learning and doing. Proceedings of ICLS 96.

[19] Ewing S. C (1998). Alternative assessment: Popularity, pitfalls, and potential. Prog. Trends Pract. Higher Educ.

[20] Worthen B (1993). Critical issues that will determine the future of alternative assessment. Phi Delta Kappan.

[21] Albanese M. A, Mitchell S (1993). Problem-based learning: A review of literature on its outcomes and implementation issues. Acad. Med.

[22] One Health, Center for Disease Control and Prevention.

[23] One Health Global Network.

[24] One Health, OIE.

[25] Hunter M, Donnelly C, Smart D, Smyth B, Menzies F, Hedderwick S (2014). Brucellosis in people with occupational cattle exposure in Northern Ireland: Clinical features of 53 cases. J. Infect.

[26] Ducrotoy M. J, Ammary K, Ait Lbacha H, Zouagui Z, Mick V, Prevost L, Bryssinckx W, Welburn S. C, Benkirane A (2015). Narrative overview of animal and human brucellosis in Morocco: Intensification of livestock production as a driver for emergence?. Infect. Dis. Poverty.

[27] Goksugur S. B, Bekdas M, Gurel S, Tas T, Sarac E. G, Demircioglu F, Kismet E (2015). An interesting case of childhood brucellosis with unusual features. Acta Clin. Croat.

[28] Kracalik I. T, Abdullayev R, Asadov K, Ismayilova R, Baghirova M, Ustun N, Shikhiyev M, Talibzade A, Blackburn J. K (2015). Human brucellosis trends: Re-emergence and prospects for control using a one health approach in Azerbaijan (1983-2009). Zoonoses Public Health.

